# On the Use of Persian Gum for the Development of Antiviral Edible Coatings against Murine Norovirus of Interest in Blueberries

**DOI:** 10.3390/polym13020224

**Published:** 2021-01-11

**Authors:** Niloufar Sharif, Irene Falcó, Antonio Martínez-Abad, Gloria Sánchez, Amparo López-Rubio, María José Fabra

**Affiliations:** 1 Department of Preservation and Food Safety Technologies, IATA-CSIC, Avda. Agustin Escardino 7, 46980 Paterna, Valencia, Spain; sharif.nilou@yahoo.com (N.S.); ferrando.falco@uv.es (I.F.); conaba@iata.csic.es (A.M.-A.); gloriasanchez@iata.csic.es (G.S.); amparo.lopez@iata.csic.es (A.L.-R.); 2 Department of Microbiology and Ecology, University of Valencia. Av. Dr. Moliner 50, 46100 Burjassot, Valencia, Spain

**Keywords:** antiviral coatings, blueberries, Persian gum, physicochemical properties

## Abstract

In the last decades, berries have been identified as important vehicles for the transmission of foodborne viruses and different strategies are being explored to eliminate or reduce viral contamination in these fruits. The aim of this work was to develop novel edible coatings with antiviral properties for inactivating and reducing murine norovirus (MNV). Firstly, the effect of gelatin (G) addition on Persian gum (PG) films was studied in terms of microstructural, mechanical, optical, and water barrier properties. The following PG:G ratios were considered: 100:0, 75:25, 50:50, 25:75, and 0:100. Microstructure analysis revealed the compatibility of both hydrocolloids since no phase separation was observed. The addition of G to PG films provided stiffer and more deformable films than pure PG, with lower water vapor permeability values. Specifically, films prepared with 50:50 PG:G ratio presented better mechanical and barrier performance. Interestingly, pure PG showed antiviral activity on murine norovirus, probably due to the presence of some impurities (mainly tannins). Adding allyl isothiocyanate (AITC) enhanced the PG antiviral activity at refrigerated temperatures in blueberries, not being affected by the AITC concentration. This effect was not observed at ambient temperature, probably due to the volatilization of AITC.

## 1. Introduction

The growing demand by consumers for healthier and more environmentally friendly foods has boosted research in the development of more sustainable strategies to extend their shelf life. One of the most plausible and widespread alternatives in the last decades is the use of edible coatings that contribute to reduce packaging material wastage. In fact, the edible-coating market for fresh fruits and vegetables has grown due to the increased use of green strategies to minimize the use of chemical preservatives. Furthermore, edible films and coatings can be used as a vehicle for active ingredients, contributing to enhance not only the food appearance but also the quality and safety of foods [1,2,3].

Edible coatings, which are usually made of natural components including proteins (i.e., gelatin) and polysaccharides (i.e., starch and gums), need to comply not only with the generally recognized as safe (GRAS) status but also they need to be acceptable by the consumer. Specifically, in recent years, exudate (i.e., arabic or acacia) and seed (i.e., guar and locust bean) gums have become a source of interest in edible-coating applications for fruits and vegetables. Gums present a high ability to attract and bind water with limited caloric contribution (acting as a fiber) and excellent film-forming ability [4]. 

An interesting, exuded gum scarcely studied as film-forming material is Persian gum (PG) (also known as Zedo gum), an anionic gum from the trunk and branches of the wild almond tree being mainly native to Iran [5]. PG naturally consists of both water soluble (ca. 30% *w*/*w*) and insoluble fractions (ca. 70% *w*/*w*). The gum’s molecular weight (Mw) varies from 4.74 × 10^6^ Da for white fractions to 2.59 × 10^6^ Da for red ones [6]. It is available in different colors (white, light yellow, dark yellow, light brown, dark brown, amber, or red) depending on the tannin content of the gum, which is influenced by the growing region [7]. Several health benefits have been associated to tannins, although their content is sometimes a restricting parameter for its application in food and pharmaceuticals since they contribute directly to major organoleptic properties such as astringency and bitter taste. In the case of edible coatings, tannins could provide an additional functionality to the edible films and coatings since tannin extracts have been reported to have antioxidant and antimicrobial properties [8,9,10].

However, even though most polysaccharides have good barrier properties at low relative humidity conditions, the large number of hydrophilic groups in their structure able to establish hydrogen bonds with water molecules limits their applicability at high relative humidity since barrier properties are detrimentally affected because of the plasticizing effect of water molecules, and, consequently, edible films or coatings become sticky. In order to broaden the range of applicability of PG-based edible coatings, blends with gelatin were proposed in this work as a strategy to reach an appropriate balance of the physicochemical properties and to assess the applicability of the developed edible coatings in fresh fruits. This is the case of fresh blueberries, an appreciated fruit for its rich composition in bioactive compounds such as flavonoids, phenolic acids, tannins, and anthocyanins. However, blueberries are highly perishable and susceptible to rapid spoilage because of microbial decay, mechanical damage, and moisture and nutritional loss [11,12], having a very short shelf life, which varies between 10 and 40 days. In fact, one of the main issues when exporting blueberries is moisture loss (measured as weight loss), which has been associated with a decrease in firmness [13]. Another important aspect in berries’ consumption is associated to the increase in the incidence of foodborne illnesses since they have been identified as important vehicles for the transmission of human noroviruses [14]. For instance, since January 2020, of the 59 alert notifications involving viruses reported in the European Union’s Rapid Alert System for Food and Feed (RASFF) database, nine were associated with berries, namely, strawberries, raspberries, blueberries, blackberries, currants, and cherries.

The use of edible coatings incorporating active agents represents an alternative way of preservation because they can actively inhibit the growth of fungi and pathogenic bacteria, and, most recently, they have also demonstrated potential to prevent contamination with human enteric viruses [2,15]. Allyl isothiocyanate (AITC) is a volatile and aliphatic sulfur-containing compound with strong and well-known antimicrobial activity [16], but its antiviral effect against foodborne viruses has not been reported yet.

Therefore, the main goals of this study were first to develop and characterize edible films based on mixtures of PG and gelatin. Then, the antiviral efficacy of the optimized PG:G ratio when applied onto the surfaces of blueberries was assessed in order to assess if the presence of residual tannins in the PG confer them antiviral activity against murine norovirus (MNV), a human norovirus surrogate. The synergistic effect of the biopolymer matrices with allyl isothiocyanate (AITC) was also evaluated. 

## 2. Materials and Methods 

### 2.1. Materials

Persian gum (PG) was purchased from a local store in Shiraz (Fars Province, Iran). Gelatin (G) from porcine skin, with reported gel strength of 180 g Bloom, was supplied by Gelita AG (Eberbach, Germany). Glycerol, chosen as a plasticizer, was provided by Panreac Química, S.A. (Castellar Del Vallés, Barcelona, Spain). Allyl isothiocyanate (AITC) (purity > 95%) was purchased from Merck-Sigma (Madrid, Spain). 

### 2.2. Preparation and Characterization of Films

Initially, 1% (*w*/*v*) of PG powder was dispersed in 100 mL of deionized water and stirred overnight at 20 °C. The suspension was centrifuged for 20 min at 4000× *g*. After removing the insoluble phase, the purified PG solution was freeze-dried and then kept at 4 °C until further use. Then, five different dispersions were prepared using PG, G, and glycerol as plasticizer. Two percent (*w*/*w*) PG was dispersed at 40 °C in deionized water under magnetic stirring. Gelatin was dissolved directly in deionized water (2% *w*/*w*) at 40 °C. Afterwards, both hydrocolloids were mixed to obtain dispersions with PG:G ratios of 100:0, 75:25, 50:50, 25:75, and 0:100. Once the blends were prepared, a fixed amount of glycerol was added (ratio hydrocolloid(s):glycerol was 1:0.30). Afterwards, dispersions were homogenized for 3 min at 13,500 rpm. Film-forming dispersions of hydrocolloids and glycerol, containing 1.5 g of total solids, were gently spread over a levelled Teflon plate (15-cm diameter) and dried at 25 °C and 45% relative humidity (RH) for 24 h. Under these conditions, flawless films were peeled intact from the casting plate. Film thickness was measured in triplicate using a hand-held digital micrometer (Palmer-Comecta, Spain, ±0.001 mm) and the average value was used in tensile properties and water vapor permeability (WVP) calculations.

Active coatings were prepared by adding the required amount of AITC to the 50:50 PG: G dispersions to reach a final concentration of 0.5% and 1% (*v*/*w*).

#### 2.2.1. Scanning Electron Microscopy (SEM)

The microstructural analysis of the films was carried out by SEM using a scanning electron microscope (Hitachi S-4800, Hitachi, Tokio, Japan) at an accelerating voltage of 10 kV and a working distance of 8–10 mm. Films were frozen in liquid nitrogen and cryo-fractured to observe the cross section of the samples. Then, they were mounted on M4 Aluminum Specimen Mount and fixed on the support using double-sided adhesive tape and, finally, a thin coating of palladium-gold was sprayed on their surface to explore the cross section of the samples.

#### 2.2.2. Transparency

The transparency of the edible films was determined, in triplicate, through the surface reflectance spectra from 400 to 700 nm in a spectrocolorimeter CM-3600d (Minolta Co, Tokyo, Japan) with a 10-mm illuminated sample area and using D65 illuminant/10 observer. The transparency was determined by applying the Kubelka–Munk theory for multiple scattering to the reflection spectra. Transparency (Ti) was calculated from the reflectance of the sample layer on a white background of known reflectance and on an ideal black background [2].

#### 2.2.3. Mechanical Properties

A Mecmesin MultiTest universal test machine (Landes Poli Ibérica, S.L., Barcelona, Spain) equipped with a 100-N static load cell was used to measure the tensile properties, according to American Society for Testing and Materials (ASTM) standard method D882-09 18 [17]. Tensile strength (TS), elastic modulus (E), and elongation at break (EAB) values were determined from the stress–strain curves estimated from force-deformation data for the different films (1 cm wide × 8 cm long). After drying, three samples of each obtained film were selected for the tensile property measurements and they were equilibrated for four days at 54% relative humidity (RH) in a cabinet using a magnesium nitrate saturated solution at 23 ± 2 °C. Equilibrated specimens were mounted in the film extension grips and stretched at 50 mm min^−1^ until breaking. The experiments were carried out 54% RH and 24 °C. Six replicates of each film formulation were tested.

#### 2.2.4. Water Vapor Permeability (WVP)

The gravimetric method based on ASTM E96/E96M-10 [18] was used to determine WVP at 23 ± 2 °C and 54–100% RH gradient. Prior to the test, the thickness of the samples was randomly measured at four points. Payne permeability cups of 3.5 cm in diameter (Elcometer SPRL, Hermelle/s Argenteau, Belgium) were filled with 5 mL of distilled water (100% RH) and then circular film samples (35-mm diameter) were secured with the outwards-facing side in contact with the air during drying. The cups were placed in pre-equilibrated cabinets at 54% RH using a magnesium nitrate saturated solution (Panreac Quimica, SA, Barcelona, Spain) and they were periodically weighted (±0.00001 g) until the steady state was reached. The free film surface during film formation (air side) was exposed to the lowest relative humidity to simulate the actual application of the films in high water activity products when stored at intermediate relative humidity. Cups with aluminum samples were used as control samples to estimate solvent loss through the sealing. Water vapor permeation rate was calculated from the steady state permeation slopes obtained from the regression analysis of weight loss data vs. time, and weight loss was calculated as the total cell loss minus the loss through the sealing. WVP was obtained by multiplying the permeance by the average film thickness. Four replicates per formulation were made. 

#### 2.2.5. Contact Angle Measurements

Contact angle measurements were carried out at ambient conditions in a Video-Based Contact Angle Meter model OCA 20 (DataPhysics Instruments GmbH, Filderstadt, Germany). Contact angle values were obtained by analyzing the shape of a distilled water drop after it had been placed over the film for 15 s. Image analyses were carried out by SCA20 software.

#### 2.2.6. Water Uptake

The water uptake of the films was estimated, in triplicate, from sorption experiments at 25 °C and 100% RH by means of weight gain using a Precisa Gravimetrics AG SERIES 320XB analytical balance (Dietikon, Switzerland). Square specimens with a total surface area of 6.25 cm^2^ were cut from the films and their initial weight was registered. 

### 2.3. Antiviral Activity of AITC

#### 2.3.1. Virus Propagation and Cell Line 

MNV-1 was propagated and assayed in RAW 264.7 cells (both kindly provided by Prof. H. W. Virgin, Washington University School of Medicine, USA). The virus stock was produced by infecting the cell lines during two days, followed by three thaw cycles and centrifugation at 660× *g* for 30 min. Infectious viruses were enumerated by determining the 50% tissue culture infectious dose (TCID50) using the Spearmen–Karber method.

#### 2.3.2. Antiviral Activity of AITC in Suspension

A first assay was carried out to elucidate the antiviral effect of AITC against murine norovirus (MNV). With this aim, a MNV suspension (about ca. 6 log TCID50/mL) was mixed in equal volumes with AITC at concentrations of 0.1% or 0.5%. Solutions were incubated at 37, 25, or 10 °C during 16 h in a bath shaker (100 rpm). The reactions were neutralized by adding Dulbecco’s Modified Eagle’s Medium (DMEM) high glucose with 10% fetal bovine serum (FBS). Viruses were recovered and 10-fold dilutions of the samples were inoculated into confluent RAW cells in 96-well plates. Then, infectious viruses were enumerated by cell culture assays as described above. The decay of MNV titers was calculated as log10 (N_t_/N_0_), where N_0_ is the infectious virus titer for untreated sample and N_t_ is the infectious virus titer for AITC-treated samples [19]. Positive controls were virus suspensions in phosphate buffered saline (PBS) under the same experimental conditions. Each treatment was performed in triplicate.

### 2.4. Challenge Tests

#### 2.4.1. Surface Solid Density (SSD) 

Selected blueberries (*Vaccinium corymbosum*) were dipped in the coating solution for 2 min and air-dried for 1 h at room temperature. The mean value of the coating was calculated in 10 samples by quantifying the SSD, as described by Falcó et al. [15] (Equation (1)).

SSD = (MCA⋅X_s_)/A_s_ (1)

where MCA is the mass of coating solution adhered to the blueberry surface, Xs is the mass fraction of solids present in the film-forming solution (FFD), and A_s_ is the average sample surface area. The average sample surface area (A_s_) was estimated by considering blueberry geometry as a sphere with a known height (measured in triplicate using a digital micrometer) and volume (measured with a pycnometer, using water as reference liquid). Samples were weighed before and after coating to determine the mass of coating solution adhered to the fruit surfaces (MCA). The non-coated sample was used as a control.

#### 2.4.2. Antiviral Test on Blueberries

Blueberries were exposed to UV for 15 min in a flow safety cabinet. Then, berries were inoculated by spraying 50 μL of MNV suspension (ca. 6 and 4 log TCID50/mL) and dried under continuously circulating laminar flow for 1 h at room temperature before application of the coating treatments. Each blueberry was dipped in the coating solution, i.e., PG:G:Allyl (0.5%) and PG:G:Allyl (1%) for 2 min. After letting it dry, individual samples were placed into sterile tubes and incubated at 10 and 25 °C. After 24 h, individual untreated and treated blueberry samples were placed in a tube containing 5 mL of DMEM supplemented with 10% fetal bovine serum (FBS) and shaken for 2 min at 180 rpm. Finally, berries were removed from the tube and serial dilutions were performed from the resultant virus suspension. Each treatment was performed in triplicate. Positive controls were uncoated berries and coated berries without AITC using different formulations (i.e., G coating, PG coating, and 50:50 PG:G coating) in its formulation under the same experimental conditions. The decay of MNV titers was calculated as log10 (N_x_/N_0_), where N_0_ is the infectious virus titer for control films and N_x_ is the infectious virus titer for films containing AITC [18].

### 2.5. Statistical Analysis

Data of each test were statistically analyzed. The statistical analysis was carried out by means of IBM SPSS Statistics software (v.23) (IBM Corp., New York, NY, USA) through the analysis of variance (ANOVA). Comparison of the means was done employing the Tukey’s Honestly Significant Difference (HSD) at the 95% confidence level. All data are presented as mean ± standard deviation.

## 3. Results

### 3.1. Characterization of Pure Persian Gum (PG) Films and Their Blends with Gelatin (G)

**Figure 1:**
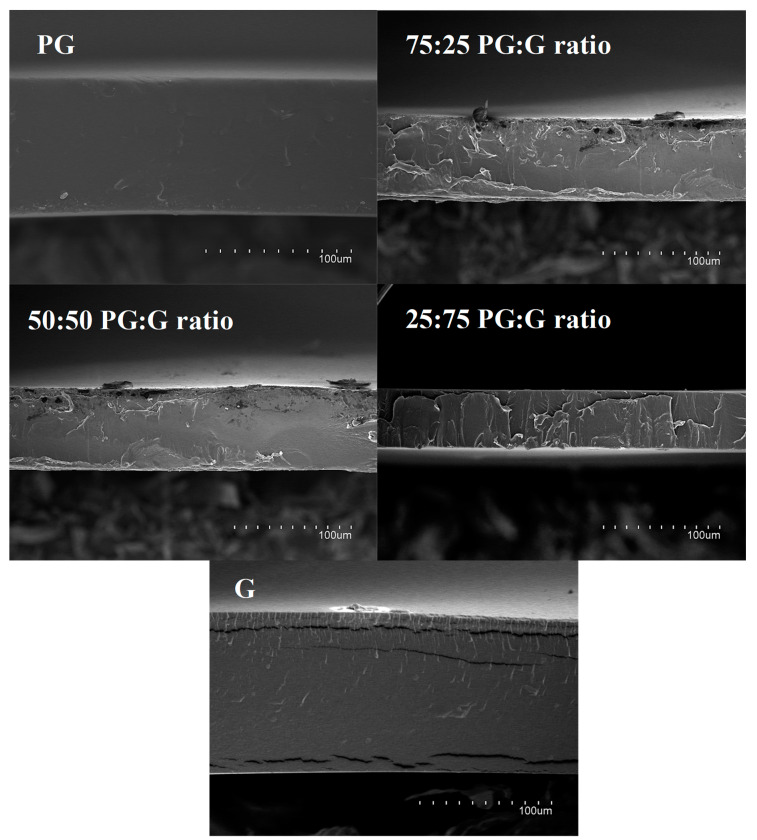
SEM micrographs of the cross sections of the developed films using neat Persian gum (PG), gelatin (G), and their combinations.

Edible coatings have to achieve different characteristics for successfully being applied in a food product. In this work, aqueous suspensions of PG and G were used to develop films by mixing them at different ratios (100:0, 75:25, 50:50, 25:75, and 0:100 PG:G ratio), and the resulting microstructure of the edible films was analyzed to better understand the internal organization of both hydrocolloids and their impact on the physicochemical properties of the films. Thus, the morphology of the films’ cross sections was evaluated by SEM and representative images are shown in Figure 1. As observed, pure PG or G films showed a smooth and homogeneous aspect with a continuous phase in the biopolymer matrix, whereas PG:G blended films exhibited a rougher microstructure. On the microstructural level, pure G films showed brittleness, which can be related to the higher rigidity observed in these films, as it will be detailed below. This probably did not occur in wetter films since water molecules act as a plasticizer, reducing the micro-fractures produced by the electron impact during observation. In blended films, both hydrocolloids were compatible as deduced from the SEM images since no phase separation was observed. Therefore, it can be assumed that electrostatic interactions among PG, G, and glycerol favored the integration of hydrocolloids in a continuous matrix during the film-drying process. In fact, electrostatic attraction has been reported as the main driving force for the complexation between proteins and polysaccharides [20]. 

**Figure 2:**
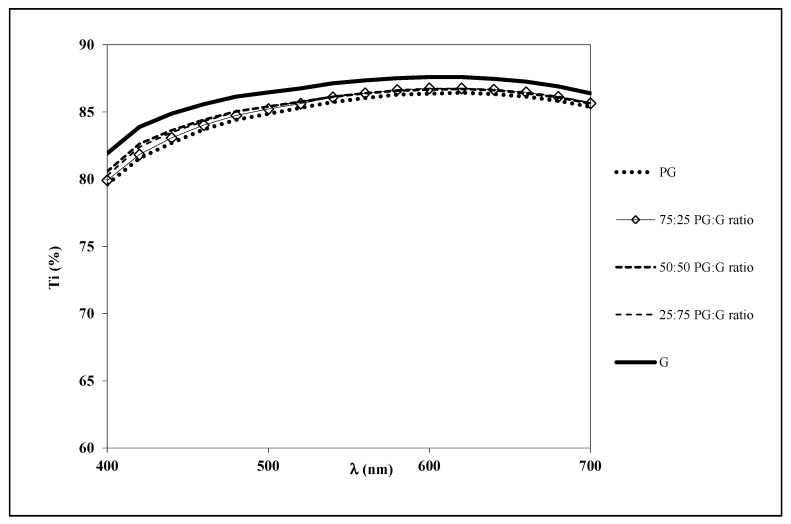
Spectral distribution of internal transmittance (Ti) of the developed edible films.

The tortuosity of the internal structure of the films usually alters the light transmission/dispersion behavior of the film and, thus, its transparency [21]. Film transparency was evaluated by means of internal transmittance (Ti) and the results are plotted in Figure 2. A similar pattern was observed for all films over the wavelength range considered. The highest Ti values were found in pure G films, whereas the incorporation of PG slightly reduced (*p* < 0.05) the internal transmittance, which reached its lowest value in films prepared with pure PG. The main differences appeared at low wavelength in PG-containing films, indicating the development of some yellowness, which can be ascribed to the presence of some impurities in the PG (such as tannins [7]). A similar trend was observed in whey protein-mesquite gum films [22]. However, differences were not significant (*p* > 0.05), and the developed films can be considered as highly transparent, which is of great interest, especially for food coating as it usually influences consumers’ acceptance of the product. 

The performance of the films was assessed in terms of mechanical and water barrier properties. As shown in Table 1, the mechanical properties, measured by means of tensile testing, were largely affected by the PG:G ratio. Pure PG films showed very poor mechanical performance with values in the range of those previously reported in literature [23,24], which could compromise their application as edible coating. However, blends with gelatin significantly improved the rigidity and strength (as suggested by the elastic modulus and tensile strength values) of the films. According to the zeta potential results of the corresponding film-forming dispersions (FFD, see Supplementary Material Table S1), this improvement can be explained by potential electrostatic interactions between both biopolymers, which promote a more cohesive network, increasing their hardness and stretchability, thus supporting the favorable effect of the gelatin in the blend films. Interestingly, it seems that the presence of these electrostatic complexes did not interfere in the gelatin entanglements when the G content was higher than 50%, thus not affecting the extensibility of the G films.

In agreement with the results found, the addition of tragacanth gum and Persian gum to gelatin films provided more flexible films than those made of gelatin [23]. Likewise, sodium caseinate and whey protein combined with Persian gum or mesquite gum, respectively, also showed greater flexibility than pure protein films [22,25].

Concerning the water vapor permeability values, with the results summarized in Table 2, there is evidence of the positive effect of preparing blend films. Pure PG films were significantly more permeable than pure gelatin. Interestingly, the obtained WVP values for blended films did not show a clear tendency with the gelatin content but a significant decrease in the 50:50 PG:G ratio films were found, which suggests a greater interaction between both biopolymers in the film matrices. WVP values were in the same range of those found in literature for neat gelatin [2,26,27]. 

The water sorption capacity of the films was also evaluated through gravimetrical tests (see Table 2). According to what was expected, pure PG films showed a greater amount of water adsorbed by the films. This can be directly related to the more hydrophilic character of gums when compared with proteins [22]. In general, a trend to reduce the water sorption was observed in gelatin-containing films, indicating that the lower permeability of the blended films was related to a reduction in the water sorption, being more obvious for those prepared with 50:50 PG:G ratio. This might be again related to both the greater barrier capacity of gelatin films compared to pure PG and the potential interactions between an oppositely charged protein–gum system. 

The water affinity was also assessed by direct measurement of contact angles of a water drop deposited on the upper surface of the films, and the results are displayed in Table 2. The estimated contact angle values evidenced that pure PG film showed a much more hydrophilic surface (contact angle ~41°). The presence of gelatin led to a marked increase in the contact angle values, being higher in films prepared with 50:50 PG:G ratio, thus suggesting that not only the gelatin fraction but also the electrostatic interactions established between both components led to a lesser amount of free -OH groups in the surface of the films. 

### 3.2. AITC-Containing Active Coatings on Blueberries

**Figure 3:**
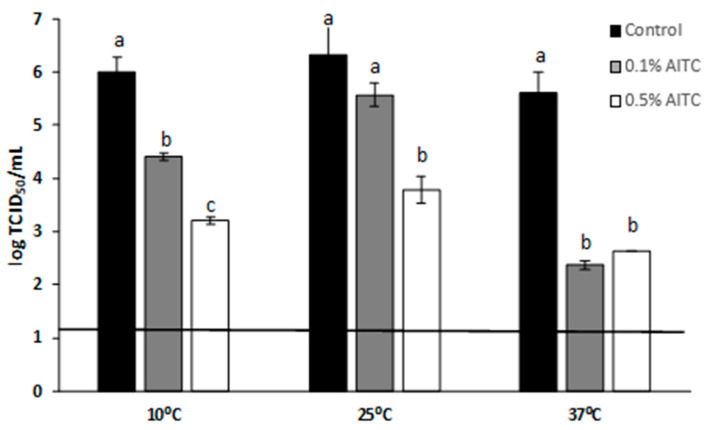
Reduction of murine norovirus (MNV) titers (log TCID50/mL) after overnight treatment at 37, 25, and 10 °C at different concentrations of allyl isothiocyanate (AITC). Each bar represents the average of triplicates. Within each temperature, different letters (a–c) denote significant differences between AITC concentration (*p* < 0.05).

In the second part of this work, antiviral edible coatings were developed by adding AITC to the biopolymeric matrices. To this end, the antiviral activity against MNV of the AITC was first analyzed at three different temperatures (10, 25, and 37 °C), and the results are displayed in Figure 3. Overall, there was a noticeably lesser reduction of MNV titers at lower temperatures. Higher reductions were reported for MNV after overnight (ON) incubation at 37 °C, decreasing titers by 3.25 log TCID50/mL and 3.00 log TCID50/mL for 0.1% and 0.5% of AITC, respectively. Statistically significant reductions (*p* < 0.05) on MNV infectivity were observed for both AITC concentrations at 10 °C (with the MNV titers being reduced by 1.58 and 2.79 log TCID50/mL after ON incubation with 0.1 and 0.5% AITC, respectively), while at 25 °C only the greatest concentration significantly reduced the virus titers. Interestingly, although antiviral activity was higher at 37 °C, this effect was not dose-dependent at 37 °C, which can be explained by the fact that this compound can be easily volatized at higher temperatures. The higher antiviral efficiency at 37 °C could be explained by the decomposition products from AITC at 37 °C [28], which could have a greater antiviral effect than the pristine compound. Similarly, a higher antiviral activity was observed for catechin derivatives (caused by degradation and epimerization reactions) than for catechin [19].

**Figure 4:**
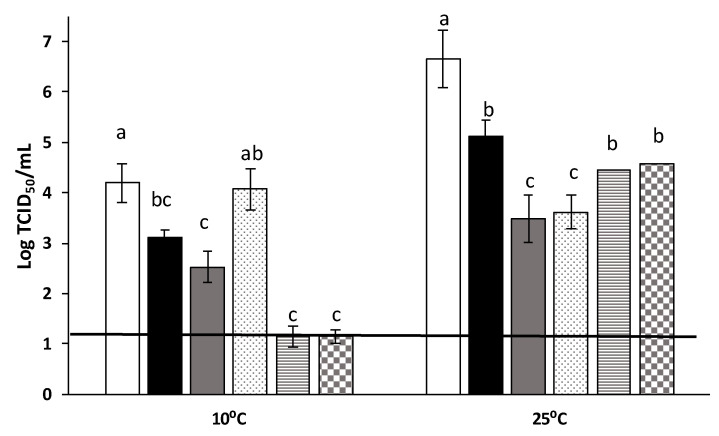
Reduction of murine norovirus (MNV) titers (log TCID50/mL) on blueberries’ surfaces after treatment coatings at two different temperatures’ storage.Each bar represents the average of triplicates. Within each temperature, different letters (a–c) denote significant differences between treatments. White bars: control without coating; black bars: G coating; grey bars: PG coating; dotted bars: 50:50 PG:G coating; dashed bars: PG:G:Allyl (0.5%); squared bars: PG:G:Allyl (1%); black line depicts the detection limit.

Subsequently, challenge tests on coated blueberries at two different temperatures (10 and 25 °C) were carried out under conditions of *in vivo* storage, mimicking realistic scenarios of fruit handing, to ascertain the virucidal effectiveness of edible coatings containing AITC. Based on the physicochemical performance and, more specifically, on the WVP of the films, 50:50 PG:G was selected to develop active films and, for comparative purposes, neat G and PG solutions were also used to coat fresh blueberries inoculated with MNV. It should be noted that the surface solid density (SSD) values, which can be used as an estimation of coating thickness, was not affected neither by the PG:G ratio or by the presence of AITC. Thus, the antiviral efficiency will be better explained by the composition of the coating rather than the deposited amount, which was ~1.52 g/m^2^. Figure 4 displays the antiviral activity against MNV after an ON incubation. Interestingly, pure PG coatings exerted antiviral activity, probably ascribed to the presence of some tannins in the pristine material that confers their brownish color [7]. Previous works have demonstrated the antiviral potential of tannins and other polyphenolic compounds [29,30]. Interestingly, the incorporation of AITC significantly reduced the MNV titers at 10 °C in comparison with the fruits coated with the formulations not containing the active compound; but no effect was observed when higher temperatures were used. This could be related to the volatilization of AITC during storage at higher temperatures; thus, being less efficient in viral inactivation. The antiviral activity of the AITC coatings was not dose-dependent, suggesting that some of the AITC was lost during coating formation. In fact, antimicrobial activity has been previously reported in essential oils-loaded films in which losses of volatile compounds were even greater than 99% [31].

## 4. Conclusions

In this work, antiviral edible coatings based on PG, G, and AITC were developed. Pure PG films showed very poor mechanical and water vapor barrier performance. In contrast, the addition of gelatin improved the mechanical and water barrier properties of pure PG films due to potential electrostatic interactions between both biopolymers, which promote a more cohesive network. The best results in terms of mechanical and water barrier properties were achieved using 50:50 PG:G ratio. This ratio was selected for preparing antiviral edible coatings and they were compared to neat G and PG coatings. 

Specifically, neat PG-based coatings showed antiviral activity against MNV, which was significantly increased by the addition of AITC at refrigerated temperatures, although it was not affected by the AITC concentrations. This may be attributed to the volatilization of AITC, which also explained the low antiviral activity of AITC at ambient temperature.

## Figures and Tables

**Table 1:** Mechanical properties (E: elastic modulus, TS: tensile strength, EAB: elongation at break) of the films obtained with Persian gum and gelatin.

PG:G Ratio	E (MPa)	TS (MPa)	EAB (%)
100:0	55 (8) ^a^	1.5 (0.2) ^a^	1.40 (0.1) ^a^
75:25	158 (8) ^b^	5.4 (2.7) ^b^	1.18 (0.04) ^a^
50:50	272 (5) ^c^	6.1 (1.3) ^c^	3.31 (0.10) ^b^
25:75	618 (41) ^d^	9.6 (1.2) ^c^	3.10 (0.32) ^b^
0:100	675 (60) ^d^	12.4 (0.7) ^c^	3.71 (0.53) ^b^

Mean value (standard deviation). Different superscript letters in each column denote significant differences (*p* < 0.05).

**Table 2:** Water vapor permeability (WVP), contact angle, and water uptake of the films obtained with Persian gum and gelatin.

PG:G Ratio	WVP (Kg-/Pa-s-m^2^) 10^13^	Contact Angle (°)	Water Uptake (%)
100:0	7.00 (0.14) ^a^	41.3 (1.4) ^a^	174 (11) ^a^
75:25	3.44 (0.30) ^b^	70.4 (1.1) ^b^	165 (11) ^a^
50:50	2.8 (0.20) ^c^	101.2 (0.7) ^c^	148 (6) ^ab^
25:75	3.35 (0.23) ^b^	98.1 (3.3) ^cd^	146 (6) ^b^
0:100	3.67 (0.30) ^b^	87.4 (2.9) ^d^	152 (5) ^ab^

Mean value (standard deviation). Different superscript letters in each column denote significant differences (*p* < 0.05).

## Data Availability

The data presented in this study are available on request from the corresponding author.

## Supplementary Materials

The following are available online at https://www.mdpi.com/2073-4360/13/2/224/s1. Table S1: Zeta potential values of the film-forming dispersions (FFD).
