# Supplementary material for: On the Use of Persian Gum for the Development of Antiviral Edible Coatings against Murine Norovirus of Interest in Blueberries
Source: Polymers (Basel). 2021 Jan 11;13(2):224. doi: 10.3390/polym13020224 (PMC7827901; doi:10.3390/polym13020224)
Supplement: Supplementary file 1 [file polymers-13-00224-s001.pdf]

Supplementary Material

On the use of Persian Gum for the development of antiviral edible coatings against Murine Norovirus of interest in blueberries

Niloufar Sharif[1], Irene Falcó[1,2], Antonio Martínez-Abad[1], Gloria Sánchez[1], Amparo López-Rubio[1], María José Fabra[1*]

Table S1. Zeta potential values of the FFD.

PG:G ratio	Zeta Potential (mV)
100:0	-13.7 (0.8)
75:25	-8.3 (0.7)
50:50	-2.5 (0.3)
25:75	1.0 (0.01)
0:100	3.9 (0.1)
